# Knockdown of copper chaperone antioxidant-1 by RNA interference inhibits copper-stimulated proliferation of non-small cell lung carcinoma cells

**DOI:** 10.3892/or.2013.2436

**Published:** 2013-04-29

**Authors:** HUAWEI CAI, FANGYU PENG

**Affiliations:** 1 Department of Radiology, University of Texas Southwestern Medical Center, Dallas, TX 75390-8542, USA; 2 Advanced Imaging Research Center, University of Texas Southwestern Medical Center, Dallas, TX 75390-8542, USA; 3 Harold C. Simmons Comprehensive Cancer Center, University of Texas Southwestern Medical Center, Dallas, TX 75390-8542, USA

**Keywords:** copper metabolism, copper chaperones, antioxidant-1, non-small cell lung carcinoma, RNA interference, cancer therapy

## Abstract

Copper is required for cell proliferation and tumor angiogenesis. Cellular copper metabolism is regulated by a network of copper transporters and chaperones. Antioxidant-1 (ATOX1) is a cytosolic copper chaperone important for intracellular copper transport, which plays a role in the regulation of cell proliferation by functioning as a transcription factor in cell growth signal-transduction pathways. The present study aimed to explore the role of ATOX1 in the copper-related regulation of lung cancer cell proliferation by immunohistochemical (IHC) analysis of ATOX1 expression in non-small cell lung cancer (NSCLC) tissue samples and by assessing the effects of RNA interference (RNAi)-mediated knockdown of ATOX1 on copper-stimulated proliferation of NSCLC cells. Overexpression of ATOX1 was detected in NSCLC by IHC analysis of the tissue samples from patients diagnosed with NSCLC when compared with expression of ATOX1 in non-malignant lung tissue samples. Knockdown of ATOX1 in the NSCLC cells transduced by a lentiviral vector encoding short hairpin RNA (shRNA) specific for ATOX1 was associated with reduction in copper-stimulated cell proliferation. These findings suggest that ATOX1 plays an important role in copper-stimulated proliferation of NSCLC cells and ATOX1 holds potential as a therapeutic target for lung cancer therapy targeting copper metabolism.

## Introduction

Cellular malignant transformation is associated with metabolic changes, and many of the molecules in signal-transduction pathways regulating cancer metabolism have been explored as a target for molecular cancer therapy (1–3). In addition to changes in glucose metabolism, cellular malignant transformation is also associated with changes in other metabolic pathways, including protein synthesis, fatty acid metabolism, and metal metabolism, such as copper metabolism. Many molecules in signal-transduction pathways regulating cancer metabolism have been investigated as potential targets for cancer gene therapy, including induction of apoptosis and tumor regression with short interfering RNA (siRNA) specific for pyruvate kinase isoform 2 (PKM2) related to glucose metabolism (4).

Copper is a co-factor required for the normal function of many enzymes involved in physiological processes such as respiration, immune response and wound repair in humans (5,6). Copper is required for cell proliferation and tumor angiogenesis (7–9). A high level of copper ions has been previously detected in tumor tissues (10,11). Human tumor xenografts with increased ^64^Cu radioactivity in mice were visualized by positron emission tomography (PET) using positron emitting copper-64 chloride (^64^CuCl_2_) as a tracer (12,13). Because excess copper is cytotoxic, intracellular copper homeostasis is tightly regulated by a delicate network of copper transporters and copper chaperones (14,15). These copper transporters and chaperones are potential targets for cancer therapy targeting copper metabolism.

Copper chaperones are small molecules responsible for intracellular copper transport (16–19), which include antioxidant 1 (ATOX1), cytochrome c oxidase 17 (Cox17), and copper chaperone for superoxide dismutase (CCS). Accumulating evidence suggests that copper chaperones may play an important role in the oncogenesis of lung cancer since: i) a high expression level of Cox17 was detected in lung cancer and Cox17 has been implied as a therapeutic target (20) and ii) ATOX1 is a cytosolic copper chaperone which plays a role in regulating cell proliferation by functioning as a transcription factor in the cell growth signal-transduction pathway (21,22). We hypothesized that ATOX1 may play a role in copper-regulated proliferation of NSCLC cells, and ATOX1 may be a new target for RNAi-based cancer therapy targeting copper metabolism.

The present study aimed to test our hypothesis by examining the expression of ATOX1 in tissue samples from patients diagnosed with NSCLC, followed by assessing the effects of the knockdown of ATOX1 by RNA interference (RNAi) on the proliferation of NSCLC cells, using NSCLC cells infected with a lentiviral vector encoding short-hairpin RNA (shRNA) specific for ATOX1.

## Materials and methods

### Chemical reagents and antibodies

Copper chloride (CuCl_2_) at a cell culture tested grade was purchased from Sigma-Aldrich (St. Louis, MO, USA). Cell lysis buffer (20 mM Tris-HCl, pH 7.5, 150 mM NaCl, 1 mM Na_2_EDTA, 1 mM EGTA, 1% Triton X-100, 2.5 mM sodium pyrophosphate, 1 mM β-glycerophosphate, 1 mM Na_3_Vo_4_, 1 μg/ml leupeptin) was from Cell Signaling Technology (Danvers, MA, USA). Anti-ATOX1, anti-Cox17 (cytochrome C-17), anti β-actin mouse monoclonal antibodies, and horseradish peroxidase (HRP)-labeled rabbit anti-mouse IgG secondary antibody were all purchased from Novus Biologicals (Littleton, CO, USA). Anti-CCS antibody was purchased from Santa Cruz Biotechnology (Santa Cruz, CA, USA).

### Cell lines and cell culture

Human non-small cell lung carcinoma (NSCLC) cell lines (A549, H460, H1299, H1355, H1703 and SKLU-1) were purchased from ATCC (Manassas, VA, USA). The cells were cultured in Dulbecco’s modified Eagle’s medium (Gibco-BR, Grand Island, NY, USA), with 10% fetal bovine serum purchased from Atlanta Biologicals (Lawrenceville, GA, USA), 100 U/ml penicillin, and 100 mg/ml streptomycin purchased from Biosource International (Camarillo, CA, USA). Antibiotic G418 was purchased from Sigma. For copper-stimulated cell proliferation assay, cells were cultured in DMEM medium containing 2% fetal bovine serum (FBS) supplemented with various doses of CuCl_2_.

### Construction of plasmid vectors encoding ATOX1 shRNA

To prepare plasmid vectors encoding shRNA specific for ATOX1 (pATOX1-shRNA), ATOX1 shRNA sequences were designed and cloned downstream of the SP3 promoter in a plasmid vector from SABiosciences, Inc. (Frederick, MD, USA), using a method specified by the manufacturer. Scramble RNA sequence with no homology to any human mRNA by BLAST search (National Center for Biotechnology Information, NIH, Bethesda, MD, USA) was used for construction of a control plasmid vector (pSCR-shRNA). The nucleotide sequence of ATOX1-shRNA and SCR-shRNA in the plasmid vector was confirmed by sequencing prior to use for testing the knockdown of ATOX1 in NSCLC cells. Efficacy of ATOX1 shRNA for knockdown of ATOX1 expression was evaluated by immunoblot assay of cell lysates of SKLU-1 NSCLC cells (ATCC) stably transfected with the pATOX1-shRNA plasmid vector DNA after selection of G418-resistant stable transfected cells.

### Construction of lentiviral vectors encoding AOTX1 shRNA

For constant suppression of ATOX1 expression in NSCLC cells, a lentiviral vector encoding ATOX1 shRNA (Lenti-ATOX1-shRNA) was constructed for knockdown of ATOX1, in a method similar to that previously described (23,24). Briefly, the ATOX1 shRNA was designed, and the efficacy of ATOX1 shRNA for knocking down the expression of ATOX1 was determined by western blot analysis, using cell lysates of the NSCLC cells transfected with the plasmid vectors containing different ATOX1 shRNA sequences. Subsequently, pLenti-ATOX1-shRNA vector was prepared by cloning of an ATOX1 shRNA sequence with confirmed efficacy for knocking down expression of ATOX1 at the *Bam*H1 and *Eco*R1 site of a pGreenPuro lenti-shRNA expression vector from System Biosciences (Mountain View, CA, USA). To prepare the lentivirus encoding ATOX1 shRNA, 293 cells were transfected with a mixture of pLenti-ATOX1-shRNA and lentiviral packaging plasmid DNA. At 72 h post-transfection, the supernatant containing lentivirus particles was harvested, and the titers (pfu/ml) of the Lenti-ATOX1-shRNA virus were determined using the UltraRapid Lentiviral Titer kit from System Biosciences. A stock solution of the Lenti-ATOX1-shRNA virus was stored at −80°C for use within 6 months post-preparation.

### Western immunoblot assay

NSCLC cells were infected with the Lenti-ATOX1-shRNA virus and subjected to puromycin selection to obtain the cells in which expression of ATOX1 was constantly suppressed. Western immunoblot assay was performed to examine the expression of ATOX1 in the NSCLC cells infected with Lenti-ATOX1-shRNA, in a method similar to that previously described (25). Briefly, cell lysates were prepared using cell lysis buffer (Cell Signaling Technology), and the total protein concentration of the supernatant was determined using the Bio-Rad BCA protein assay kit (Hercules, CA, USA). Following separation of cellular proteins (30–50 μg) by SDS-PAGE (12%) and transfer onto a PVDF membrane, the blots were blocked in 5% de-fatted milk powder in PBST (130 mM NaCl, 10 mM NaH_2_PO_4_, pH 7.4, 0.1% Tween-20) at 4°C overnight. The blocked blots were incubated with mouse anti-ATOX1 monoclonal antibody (1:1,000 dilution) at 37°C for 1 h, followed by washing with PBST and incubation with HRP-conjugated rabbit anti-mouse secondary antibody (Novus) in 5% milk/PBST at room temperature for 1 h. Immunoreactivity on the blots was visualized by chemiluminescence using a Supersignal Western Blot Enhancer kit (Pierce, Rockford, IL, USA). Following stripping of the blots using a blot stripping buffer from Pierce, the blots were reprobed with anti-β actin antibody (1:10,000) for visualization of the sample loading control. Semi-quantitative analysis of the western immunoblot was conducted by densitometry using AlphaEase FC™ software (Alpha Innotech, Santa Clara, CA, USA), in a method modified from that previously described (26). The integrated density value (IDV) measured from ATOX1 and β-actin bands on blots was used for calculation of the percentage of ATOX1 suppression or silencing in the cells transfected with the plasmid DNA encoding shRNA targeting ATOX1 or the cells transduced by the lentiviral vector encoding shRNA targeting ATOX1, relative to ATOX1 expression in wild-type cells. Briefly, IDV of the β-actin band for a test sample was normalized to IDV of the β-actin band of the wild-type cell sample to minimize variation in the protein loading controls. A ratio of ATOX1 expression in the cells transduced with ATOX1 shRNA vectors relative to the ATOX1 expression in wild-type cells was calculated by dividing IDV of the ATOX1 band from the cells transduced with ATOX1 shRNA vectors with IDV of the ATOX1 band from the wild-type cell lysates. Subsequently, the normalized ratio of ATOX1 expression was obtained following normalization of the ATOX1 expression ratio against the β-actin loading control. Finally, the percentage of ATOX1 suppression or silencing was calculated as (1 − normalized ATOX1 expression ratio × 100%).

### Cell proliferation assay

Cell proliferation assay was conducted using an MTT cell proliferation assay kit purchased from ATCC (Manassas, VA, USA), using the protocol specified by the manufacturer based on a previously described method (25,27). Briefly, cells were inoculated into a 96-well plate (1×10^3^ cells/well) and cultured in DMEM without serum under serum starvation conditions for 24 h. Subsequently, the cells were incubated with DMEM supplemented with CuCl_2_ dissolved in PBS (10 μM) at 37°C for up to 72 h. At 24 h post inoculation, MTT agent solution was added, and the optical density (OD) was measured at a wavelength of 570 nm with a reference wavelength of 630 nm. The cell numbers at the time of the MTT assay were recorded as percentages of the cell numbers initially inoculated. The experiment was conducted in triplicate for each time point and repeated three times.

### Immunohistochemical analysis

Immunohistochemical (IHC) analysis of copper chaperone expression in tumor tissue samples was conducted using a a previously described method (12,13), according to a protocol approved by the Institutional Review Board, University of Texas Southwestern Medical University at Dallas, Texas. Briefly, formalin fixed, paraffin-embedded, archived tissue samples from the tissue bank were subjected to de-paraffining and antigen retrieval by steam cooking for 5 min in citrate at pH 8.0. After quenching of the endogenous peroxidase and blocking with 5% BSA for 30 min, sections were incubated with mouse monoclonal anti-ATOX1 antibody (1:200), anti-Cox17 antibody (1:200), or anti-CCS antibody (1:200), at room temperature for 2 h, followed by visualization of immunoreactivity with HRP-labeled rabbit anti-mouse IgG secondary antibody (ABC method). Immunoreactivity of copper chaperones (ATOX1, CCS or Cox17) was examined under a microscope. Semi-quantitative analysis of ATOX1 expression in tissue samples was conducted by visual assessment of the intensity of immunoreactivity on tissue samples, assuming that the intensity of immunoreactivity was correlated with the level of expression. Intensity of immunoreactivity was scored visually and recorded as following: 3 + for strong, 2+ for moderate, 1+ for weak immunoreactivity, and 0 for negative immunoreactivity.

### Statistical analysis

The percentages of cells determined from the MTT assay are expressed as means ± SD. In order to determine whether growth of the cells of two groups (Lenti-ATOX1-shRNA vs. Lenti-SCR-shRNA cells) differed between the three time-points (24, 48 and 72 h), a (2 × 3) mixed design ANOVA was applied, where the between-subject factor was the group (with 2 levels) and the within-subject factor was time (with 3 levels). These tests were applied for MTT assays in the presence and absence of CuCl_2_ at the indicated concentration. If a significant (time × group) interaction was determined for each of the comparisons, it was followed up with an independent sample t-test for each of the 3 time points (24, 48 and 72 h). A P-value of <0.05 was considered to indicate a statistically significant result.

## Results

### Overexpression of ATOX1 in NSCLC tissues by IHC analysis

**Figure 1:**
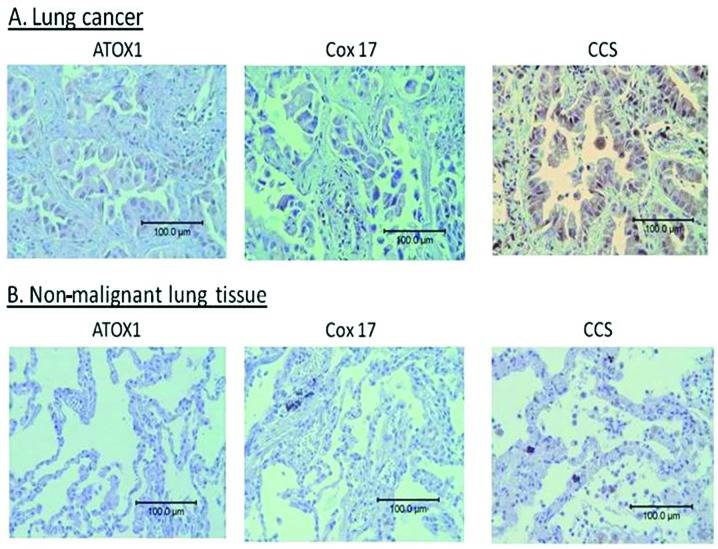
Expression of copper chaperones in NSCLC by immunohistochemical (IHC) analysis. (A) Representative IHC analysis of the expression of ATOX1, Cox17 and CCS in NSCLC tissue samples. (B) Representative IHC analysis of expression of ATOX1, Cox17, and CCS in non-malignant lung tissue samples. A high level of expression of ATOX1 and Cox17 was detected in NSCLC tissue samples, when compared with the expression of these two copper chaperones in the control non-malignant lung tissue samples. In contrast, a high level of expression of CCS was detected in the NSCLC and non-malignant lung tissue samples. Scale bar, 100 μM. ATOX1, antioxidant 1; Cox17, cytochrome c oxidase 17; CCS, copper chaperone for superoxide dismutase.

Differential overexpression of ATOX1 was detected in all of the NSCLC tissue samples (n=5) tested in this study (Fig. 1A and Table I), when compared with the low level of expression of ATOX1 in the non-malignant lung tissue (n=3) of the control group (Fig. 1B and Table I). In contrast, a high level of expression of Cox17 was detected in only 3 out of 5 NSCLC tissue samples (Table I). A high level of expression of CCS was detected in all of the NSCLC tissue samples (n=5) and non-malignant lung tissue samples (n=3), as shown in Table I.

### Variable expression of copper chaperones in NSCLC cells by immunoblot assay

**Figure 2:**
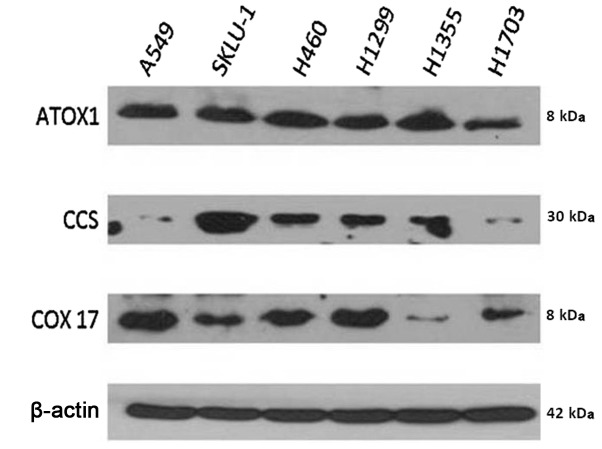
Expression of copper chaperones in NSCLC cells by western immunoblot assay. Expression of ATOX1 was detected in all of 6 NSCLC cell lines, at a similar expression level. Expression of Cox17 was variable, with the expression level of Cox17 in the H1355 cell lines lower than levels in the other cell lines. Expression of CCS in NSCLC cells was also variable, with its expression levels in A549 and H1703 lower than levels in the other 4 NSCLC cell lines. β-actin was used as a loading control.

Expression of copper chaperones (ATOX1, CCS and Cox17) in 6 established NSCLC cell lines was examined by western immunoblot assay (Fig. 2). Expression of ATOX1 was detected in all 6 established NSCLC cell lines, with similar abundance. In contrast, expression of Cox17 was variable, with a higher expression level in the A549, H460 and H1299 cell lines compared with that detected in the other 3 NSCLC cell lines (SKLU-1, H1703 and H1355). In contrast, the expression level of CCS in the SKLU-1, H460, H1299 and H1355 NSCLC cell lines was higher than that in the other 2 NSCLC cell lines (A549 and H1703).

### Knockdown of ATOX1 expression in NSCLC cells transduced by Lenti-ATOX1-shRNA virus

**Figure 3:**
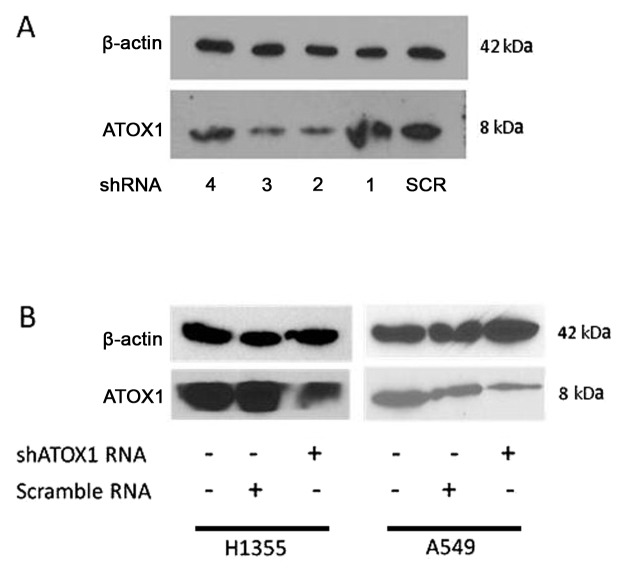
RNAi-mediated knockdown of ATOX1 in NSCLC cells by western immunoblot assay. (A) Knockdown of ATOX1 in the SKLU-1 NSCLC cells transfected with pATOX1-shRNA plasmid vector #2 and #3 as determined by western blot assay, but not in the cells transfected with pATOX1-shRNA plasmid vector # 1, #4, or pSCR-shRNA plasmid (SCR). (B) Knockdown of ATOX1 in H1355 and A549 NSCLC cells infected with the Lenti-ATOX1-shRNA lentivirus encoding the ATOX1 shRNA sequence derived from pATOX1-shRNA plasmid vector #2, but not in the wild-type cells, and the H1355 or A549 cells infected with Lenti-SCR-shRNA virus. β-actin was used as the loading control.

Four pATOX1-shRNA plasmid vectors containing shRNA sequences targeting various regions of ATOX1 mRNA (Table II) were constructed, using the shRNA plasmid vector from SABiosciences. One pSCR-shRNA plasmid vector encoding the scrambled RNA sequence (Table II) was constructed and used as a control. Variable suppression of ATOX1 was detected in the cells transfected with the pATOX1-shRNA plasmid vectors by western immunoblot assay (Fig. 3A). Semi-quantitative analysis of the western immunoblot assay demonstrated 77% suppression of ATOX1 expression in the cells transfected with pATOX1-shRNA plasmid vectors #2, and 74% suppression in the cells transfected with pATOX1-shRNA plasmid vectors #3, relative to ATOX1 expression in the cells transfected with the control plasmid vector encoding scrambled shRNA (SCR shRNA). A lentiviral vector encoding ATOX1 shRNA was prepared, using the ATOX1 shRNA sequence from pATOX1-shRNA #2 (Table II). A control lentivirus was also prepared, using scramble shRNA sequences (Table II). Knockdown of ATOX1 was detected in the A549 cells transduced by Lenti-ATOX1-shRNA (shATOX1-A549) and the H1355 cells transduced by Lenti-ATOX1-shRNA (shATOX1-H1355) by western immunoblot assay (Fig. 3B). Semi-quantitative analysis of the western immunoblot assay demonstrated a 75% suppression of ATOX1 expression in the shATOX1-A549 cells and a 77% knockdown of ATOX1 expression in the shATOX1-H1355 cells, relative to the ATOX1 expression in the wild-type A549 or H1355 cells. No suppression of ATOX1 was detected in the H1355 cells transduced by the lentivirus encoding scrambled shRNA (SCR-H1355), compared with ATOX1 expression in the wild-type H1355 cells. However, a 38% knockdown of ATOX1 expression was detected in the A549 cells transduced by the lentivirus encoding scrambled shRNA (SCR-A549), relative to ATOX1 expression in the wild-type A549 cells.

### Reduction in the copper-stimulated proliferation of the NSCLC cell in which ATOX1 was constantly suppressed by RNAi

**Figure 4:**
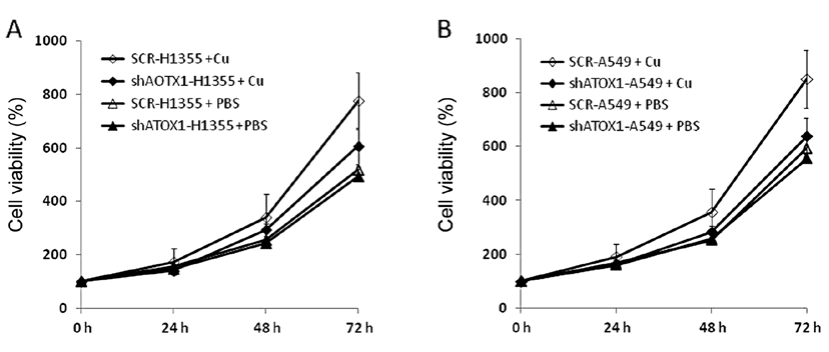
Reduction in the copper-stimulated proliferation of NSCLC cells by RNAi-mediated knockdown of ATOX1. (A) Schematic plot showing reduction in the copper-stimulated proliferation of the H1355 NSCLC cells infected with lenti-ATOX1-shRNA virus (shATOX1-H1355) cultured in medium containing CuCl_2_ dissolved in PBS buffer at a concentration of 10 μM (shATOX1-H1355 + Cu), compared with that of the control H1355 cells infected with Lenti-SCR-shRNA virus (SCR-H1355 + Cu). No significant difference was detected between the growth of the shATOX1-H1355 cells and that of the SCR-H1355 cells cultured in the medium containing no CuCl_2_ (shATOX1-H1355 + PBS and SCR-H1355 + PBS). (B) Schematic plot showing reduction in the copper-stimulated proliferation of the shATOX1-A549 cells cultured in medium containing 10 μM CuCl_2_ (shATOX1-A549 + Cu), compared with that of the control H1355 cells infected with the Lenti-SCR-shRNA virus (SCR-A549 + Cu). Again, no significant difference was detected between the growth of the shATOX1-A549 cells and that of the SCR-A549 cells cultured in medium containing no CuCl_2_ (shATOX1-A549 + PBS and SCR-A549 + PBS). Cell number (%) is the percentage of the initially inoculated cells (0 h) at the time of the MTT assay.

Effects of ATOX1 knockdown on cell proliferation were examined by comparing the proliferation of the cells in which expression of ATOX1 was constantly suppressed (shATOX1-H1355 and shATOX1-A549) with that of the control SCR-H1355 and SCR-A549 cells. In the presence of CuCl_2_ at a concentration of 10 μM, proliferation of the shATOX1-H1355 and shATOX1-A549 cells was slower than that of the SCR-H1355 and SCR-A549 cells (Fig. 4). At the end of the 72-h cell culture, the number of shATOX1-H1355 cells (625±18% of the cell number initially inoculated) was significantly less than that of the SCR-H1355 cells (775±12%) (P<0.001). Similarly, the number of shATOX1-A549 cells (639±15% of the cell number initially inoculated) was also significantly less than that of the SCR-A549 cells (851±13%) (P<0.001), at the end of the 72-h cell culture. In contrast, there was no significant difference between the number of shATOX1-H1355 cells (493±21%) and the number of SCR-H1355 cells (517±47%), at the end of the 72-h cell culture without the presence of 10 μM of CuCl_2_ in the culture medium, nor was there a significant difference between the number of shATOX1-A549 cells (554±12%) and the number of SCR-A549 cells (594±15%).

## Discussion

Copper is required for cell proliferation, angiogenesis and tumor growth (7–9). ATOX1 is a cytosolic copper chaperone required for intracellular delivery of copper to the trans-Golgi network (TGN) and other secretory compartments (14–16). Recently, it was reported that ATOX1 plays a role in the regulation of cell proliferation (21). In the present study, overexpression of ATOX1 was detected in NSCLC tissue samples, when compared with its expression in non-malignant lung tissues (Fig. 1). RNAi-mediated knockdown of ATOX1 inhibited copper-stimulated proliferation of NSCLC cells (Fig. 4). To the best of our knowledge, the data from this study for the first time demonstrated that ATOX1 plays a role in copper-stimulated proliferation of NSCLC cells. The findings of the present study shed light on the study of ATOX1 as a new target for RNAi-based NSCLC cancer therapy targeting copper metabolism.

The molecular mechanism of ATOX1 in mediating copper stimulated cell proliferation remains to be elucidated. Of note, Atox1 protein has the conserved lysine-rich region (KKTGK) at its C terminus, which may represent the nuclear localization signal. It was reported that ATOX1 also accumulates in the nucleus as a copper-dependent transcription factor to increase promoter activity of cyclin D1, an important regulator of cell cycle G1-S phase progression, and to promote cell proliferation (21,22). In addition to loss of transcriptional factor activity of cyclin D1, knockdown of ATOX1 may result in blockage of copper-related cell growth signal transduction through molecules downstream from ATOX1, such as Ccc2p homologs, and therefore a reduction in copper-stimulated cell proliferation.

Cellular copper metabolism is regulated by a network of copper transporters and chaperones. ATOX1 may interact with Cox17 and other copper chaperones in regulating copper-stimulated proliferation of NSCLC cells. RNAi-based NSCLC therapy with short-interference RNA (siRNA) targeting both ATOX1 and Cox17 may have stronger therapeutic effects on NSCLC than RNAi-based NSCLC therapy targeting ATOX1 alone. On the other hand, the efficacy of RNAi-based therapy targeting ATOX1 in NSCLC may be enhanced by concurrent use of copper-lowering anticancer drugs, such as tetrathiomolybdate. In addition to ATOX1, investigation of the potential of other copper transporters and chaperones in siRNA therapy of NSCLC and other cancers, such as ATP7B in ovarian cancer (28) and ATP7A in neuroblastoma (29) is warranted.

In summary, the data from the present study revealed the occurrence of overexpression of ATOX1 in NSCLC tissue samples, and RNAi-mediated knockdown of ATOX1 inhibited copper-stimulated proliferation of NSCLC cells. ATOX1 holds potential as a new target for RNAi-based cancer therapy of NSCLC targeting copper metabolism.

## Figures and Tables

**Table I:** Expression of copper chaperones in malignant and non-malignant lung tissues by immunohistochemical analysis.

Subject	Tissue sample	ATOX1 immunoreactivity	Cox17 immunoreactivity	CCS immunoreactivity
1	NSCLC	+++	+++	+++
2	NSCLC	+++	++	++
3	NSCLC	++	+++	+++
4	NSCLC	+++	+	++
5	NSCLC	+++	+	+++
6	Normal lung tissues	+	+	++
7	Normal lung tissues	+	+	++
8	Lung tissues with chronic inflammation	+	++	+++

Intensity of immunoreactivity: +, mild; ++, moderate; +++, strong. NSCLC, non-small cell lung cancer; ATOX1, antioxidant 1; Cox17, cytochrome c oxidase 17; CCS, copper chaperone for superoxide dismutase.

**Table II:** Oligonucleotide sequence of ATOX1-targeting shRNA and scrambled shRNA.

	Oligonucleotide sequence (up strand)
ATOX1-targeting shRNA	
#1	*TCTCG*CAACAAGAAGGTCTGCATTGA**CTTCCTGTCA**TCAATGCAGACCTTCTTGTTG*CT*
#2	*TCTCG*TGTTTCCTACCTTGGCCTTGA**CTTCCTGTCA**TCAAGGCCAAGGTAGGAAACA*CT*
#3	*TCTCG*AAGTATGACATTGACCTGCCC**CTTCCTGTCA**GGGCAGGTCAATGACATACTT*CT*
#4	*TCTCG*AATCTGAGCACAGCATGGACA**CTTCCTGTCA**TGTCCATGCTGTGCTCAGATT*CT*
Scrambled shRNA	
Control	*TCTC*GGAATCTCATTCGATGCATAC**CTTCCTGTCA**GTATGCATCGAATGAGATTCC*CT*

ATOX1, antioxidant 1; shRNA, short hairpin RNA. Letters in italics indicate the vector sequence at insertion site, underlined letters indicate sequence of shRNA insert and bold letters indicate the loop sequence.
